# Faso Food and Drug Administration (Faso FDA): strengthening regulatory systems for public health protection in Burkina Faso

**DOI:** 10.3389/fdsfr.2026.1880709

**Published:** 2026-10-14

**Authors:** Kampadilemba Ouoba, Priscilla P. M. Nyambayo, Daniel Dori, Adela Ashie, Kenneth Onu, Rasmané Semdé

**Affiliations:** 1 Laboratory of Drug Development, Joseph KI-ZERBO University, Ouagadougou, Burkina Faso; 2 Pharmacovigilance and Clinical Trials Division, Medicines Control Authority of Zimbabwe, Harare, Zimbabwe; 3 Food and Drugs Authority, Accra, Ghana; 4 African Union Development Agency-NEPAD (AUDA-NEPAD), Johannesburg, South Africa

**Keywords:** drug safety and regulation, pharmaceutical public health, good regulatory practices, developing countries, faso FDA, Burkina Faso

## Abstract

In May 2026, the Government of Burkina Faso established Faso Food and Drug Administration (Faso FDA) by Decree No. 2026-0602/PF/PRIM/MEF/MS. This unified regulatory authority mandated to oversee food products, health commodities, industrial chemicals, cosmetics, pesticides, and other products with potential public health risks. Endowed with legal personality and administrative and financial autonomy, the Agency should operate independently and mitigate risks related to corruption and conflicts of interest. Its scope aligns with that of other African national regulatory authorities such as the Ghana Food and Drugs Authority and the Rwanda Food and Drugs Authority. The legal establishment of Faso FDA is only the first step and to fully deliver on its mandate, this young regulatory authority must address six strategic priorities: securing sustainable domestic financing; strengthening governance systems and transparency; ensuring adequate infrastructure and technical capacity; developing a competent and sufficient workforce; achieving World Health Organization (WHO) Global Benchmarking Tool (GBT) Maturity Level 3; and bolstering regional and international collaboration. Addressing these priorities will position Faso FDA as a robust regulatory authority capable of safeguarding public health and promoting sustainable local production of safe, high-quality food and medical products.

## 1 Introduction

The Government of Burkina Faso adopted the Decree No. 2026-0602/PF/PRIM/MEF/MS on 22 May 2026 establishing “Faso Food and Drug Administration” (Faso FDA) (“Compte Rendu Du Conseil Des Ministres - Service d’information Du Gouvernement,” n.d.). The Agency was created through the merger of the National Agency for Health, Environmental, Food, Labour and Health Product Safety (ANSSEAT) and the National Pharmaceutical Regulatory Agency (ANRP). The ANRP coordinated the implementation of most regulatory functions, such as marketing authorisation, licensing, pharmacovigilance, market surveillance and control, regulatory inspections and the monitoring of clinical trials. The ANSSEAT, for its part, was responsible for laboratory testing and the monitoring of food and environmental risks.

The establishment of Faso FDA consolidates, under a single national authority, regulatory functions that were previously fragmented across multiple ministries, including Health, Agriculture, Trade, and Environment. According to the Council of Ministers, this reform responds to persistent challenges such as substandard and falsified medical products, cosmetics containing prohibited substances, contaminated or improperly stored food, and the widespread use of unregistered pesticides that pose risks to human health and the environment (Service d’information du Gouvernement, n.d.).

The decree establishing Faso FDA lays the foundation for its designation as a Public Health Institution with legal personality and administrative and financial autonomy. This status will enable the Agency to effectively carry out functions traditionally performed by a National Regulatory Authority (NRA), including scientific evaluation, regulatory decision-making, and post-market surveillance. However, although Faso FDA has been legally established, it is still necessary to identify the strategic priorities required to make it operational and effective. This will, in particular, enable it to support the development of local production of essential health products and priority food commodities, thereby improving access to safe, effective, and quality-assured products.

The aim of this work was to identify the key challenges that a newly established regulatory authority will face and how these can be addressed, thereby providing lessons for the future establishment of regulatory authorities in developing countries.

## 2 Scope and functions of Faso FDA

In alignment with other African regulatory authorities—including the Ghana FDA and the Rwanda FDA—Faso FDA will regulate food and beverages, medicines for human and veterinary use, blood products, medical devices, cosmetics, vaccines, pesticides, and other substances with potential public health implications (Owusu-Asante et al., 2022; Rwanda Food and Drugs Authority, n.d.).

As a comprehensive regulatory authority, its core responsibilities will include (World Health Organisation, 2024; Shabani et al., 2022):

- development and implementation of regulatory frameworks for clinical trials, manufacturing, marketing authorisation, importation, distribution, and post-marketing surveillance;
- issuance of establishment licences and authorisations for regulated professionals;
- authorisation and oversight of clinical trials;
- marketing authorisation of regulated products;
- pharmacovigilance and market surveillance;
- inspection and enforcement, including fraud prevention;
- quality control and laboratory testing;
- regulation of advertising and promotional activities;
- regional and international cooperation.

The breadth of this mandate requires strong institutional capacity, well-structured governance mechanisms, and sustainable financing strategies.

## 3 Strategic challenges and priority areas

### 3.1 Sustainable domestic financing

To fulfil its mandate, Faso FDA will require a reliable financing mechanism (Sithole et al., 2021; Ndomondo-Sigonda et al., 2020). Its legal status of administrative and financial autonomy provides a solid foundation for developing such a mechanism. The Agency’s operating budget could be supported through a combination of:

- regulatory fees (e.g., marketing authorisation, inspection, clinical trial approvals);
- levies on pharmaceutical and agri-food industries;
- earmarked taxes on sectors such as tobacco, alcoholic beverages, and energy drinks, as well as telecommunications, financial services, and mining;
- complementary funding from global health and health security partners for priority activities (Kaplan and Laing, 2003; Morgan, Yau, and Lumpkin, 2017).

As regards the collection of taxes, these must be mandated by regulation in order to be effectively implemented.

Burkina Faso has a regulatory and administrative framework governing the mobilisation, management and expenditure of public funds. This framework includes, at the legislative level, Law No. 008-2013/AN on the Code of Transparency in the Management of Public Finances in Burkina Faso; Organic Law No. 073-2015/CNT on Finance Acts and Law No. 005-2024/ALT on general regulations governing public procurement. In regulatory terms, this involves Decree No. 2019-0782/PRES/PM/MINEFID on the financial and accounting regime for State public institutions and Decree No. 2016-598/PRES/PM/MINEFID on general regulations governing public accounting. At the administrative level, the oversight of revenue and expenditure is carried out by the Board of Directors and the line ministries responsible for all state-owned public institutions. This framework will enable Faso FDA to mobilise and manage its financial resources efficiently in order to fulfil its public health mandate (Direction Générale du Budget, n.d.).

### 3.2 Good governance, institutional independence, and transparency

Governance efficiency and transparency are essential to safeguarding institutional independence (Paschke et al., 2018). Faso FDA should adopt the World Health Organization’s Good Regulatory Practices (GRP), which promote legality, consistency, impartiality, proportionality, flexibility, clarity, effectiveness, and transparency (Owusu-Asante et al., 2025).

The importance of regulatory independence has been illustrated by past public health crises. The Mediator® (*benfluorex*) scandal in France, for instance, revealed weaknesses in transparency and conflicts of interest, ultimately leading to the restructuring of the French regulatory system (Mullard, 2011). This example underscores the need for Burkina Faso to embed strong safeguards from the outset.

The introduction of specific safeguards, such as declarations of conflicts of interest by the Faso FDA staff and external experts, the publication of standards, regulatory decisions and operational procedures, and the digitisation of regulatory processes, will enable Faso FDA to achieve sound, transparent and accountable governance.

### 3.3 Human resources and workforce development

The establishment of a well-functioning NRA requires a multidisciplinary team of highly qualified professionals. Workforce development should therefore prioritise continuous training, career progression pathways, and competitive remuneration (Owusu-Asante et al., 2025).

National initiatives such as the Presidential Health Initiative and the 2025–2029 Human Resources for Health Strengthening Programme (“1000x5 Plan”) provide an opportunity to build this capacity (“Burkina Faso: Le Gouvernement Adopte Le «Plan 1000 × 5 de Développement Des Ressources Humaines En Santé» – Agence Afrique (n.d.)). With a budget exceeding USD 124.7 million and a goal of training 5,000 specialists over 5 years, this programme can support training in key regulatory areas including:

- pharmaceutical regulation and law;
- risk assessment and quality control;
- toxicology;
- quality management systems;
- information systems and data management;
- biomedical research.

Article 5 of Decree No. 2026-0602/PF/PRIM/MEF/MS establishing Faso FDA sets out provisions regarding the transfer of the assets and liabilities of the ANRP and the ANSSEAT to Faso FDA, thereby enabling better management of staff and the handover of responsibilities during the start-up phase.

### 3.4 Infrastructure and technical capacity

Faso FDA will rely on existing achievements from its precursor institutions, both of which are certified ISO 9001:2015, with ANSSEAT additionally holding an ISO/IEC 17025:2017 certification. However, further investment is essential. Adequate infrastructure–particularly laboratories, digital systems, inspection tools, pharmacovigilance platforms, high-performance databases and technology for the traceability of medical products and the detection of counterfeits–is fundamental to evidence-based decision-making and effective implementation of regulatory functions. Strong political commitment will be vital to closing current gaps.

### 3.5 Regional and international collaboration

Strategic cooperation is essential for harmonisation, regulatory convergence, and mutual recognition of decisions (Dehaghi et al., 2023). Collaboration with NRAs that have already reached ML3—such as those in Ghana, Rwanda, and Senegal—will facilitate technical support, knowledge transfer, and benchmarking. Collaboration with these sister regulatory authorities could involve adopting regulatory frameworks for reliance, and building the capacity of its technical staff through on-site training and joint assessments. Faso FDA could also benefit from their support through a clear partnership framework for the development of effective institutional development plans for the various regulatory functions.

In addition, cooperation with regional and continental bodies—including the Alliance of Sahel States (AES), West African Economic and Monetary Union (UEMOA), and the African Medicines Agency (AMA)—will contribute, through harmonisation, to addressing disparities in regulatory practices and strengthening oversight of clinical trials, inspections, pharmacovigilance, and laboratory quality control.

### 3.6 Attainment of WHO Global Benchmarking Tool (GBT) maturity level 3

Reaching WHO Maturity Level 3 (ML3) represents a critical milestone for NRAs. ML3 indicates that a regulatory system is stable, well-functioning, and aligned with international quality management principles (e.g., ISO 9004:2018). Several African NRAs—including those in South Africa, Egypt, Ghana, Nigeria, Rwanda, Senegal, Tanzania, and Zimbabwe—have achieved ML3 status (Owusu-Asante et al., 2025).

Attaining ML3 would enable Faso FDA to:

- ensure predictable and efficient regulatory processes;
- create an enabling environment for local production of medicines and vaccines;
- position Burkina Faso as a potential host for vaccine and health product manufacturing units serving regional markets.

Faso FDA will be able to draw on the results of the self-assessment carried out by the ANRP using the WHO GBT tool, and on the institutional development plans for the various regulatory functions drawn up as part of the regional work organised by the Regional Committee for the Harmonisation of Pharmaceutical Regulations of the West African Economic and Monetary Union (UEMOA) (Autorité Ivoirienne de Régulation Pharmaceutique, n.d.)

## 4 Discussion

This work highlights the establishment of a new regulatory authority for health and food products in Burkina Faso, against a backdrop of limited resources and growing challenges in public health and global health. The legal establishment of this authority–and indeed the identification of the priority challenges to be addressed–is not an end in itself, but marks the beginning of a long process of institutional development. The six priority areas described are interdependent and must be addressed holistically to enable synergy between them. For example, sustainable funding will support the development of infrastructure and the workforce; governance will promote transparency and trust; and infrastructure and cooperation will contribute to achieving maturity level 3. It is also important to emphasise that Faso FDA cannot meet these challenges and achieve sustainable institutional development without strong leadership from its senior management and firm political commitment, particularly from the Ministry of Health. Institutional leadership will foster partnership and cooperation with other highly mature African regulatory authorities such as the Ghana FDA, the Senegalese Pharmaceutical Regulatory Agency and the Rwanda FDA. As for political commitment, this will enable the accelerated implementation of Faso FDA’s endogenous self-financing mechanisms and also secure the support of regional and international partners. By addressing the six priority strategic areas outlined in this report, with the support of the Ministries of Health and Finance, Faso FDA will be able to establish itself as a strong and effective regulatory authority, capable of ensuring sustainable access to safe, high-quality health and food products. By strengthening its governance, staffing, infrastructure, funding mechanisms and regional integration, the Agency will play a vital role in protecting public health and will promote the development of local production capacity for health products and foodstuffs, with a view to achieving health and food sovereignty for Burkina Faso.

## Data Availability

The original contributions presented in the study are included in the article/supplementary material, further inquiries can be directed to the corresponding author.

## References

[B1] Agence Afrique (n.d.). Burkina Faso: le Gouvernement Adopte Le «Plan 1000×5 de Développement Des Ressources Humaines En Santé» – Agence Afrique. Available online at: https://www.agenceafrique.com/61757-burkina-faso-le-gouvernement-adopte-le-plan-1000x5-de-developpement-des-ressources-humaines-en-sante.html (Accessed August 2, 2026).

[B2] Autorité Ivoirienne de Régulation Pharmaceutique (AIRP) (n.d.). Réunion annuelle du Comité Régional pour l'Harmonisation de la Réglementation Pharmaceutique des États membres de l'UEMOA, AIRP. Available online at: https://airp.ci/posts/actualites/reunion-annuelle-du-comite-regional-pour-lharmonisation-de-la-reglementation-pharmaceutique-des-etats-membres-de-luemoa/31 (Accessed August 2, 2026).

[B3] Dehaghi R. O. A. Broojerdi A. K. Paganini L. Sillo H. B. (2023). Collaborative training of regulators as an approach for strengthening regulatory systems in LMICs: experiences of the WHO and swissmedic. Front. Med. 10, 1173291. 10.3389/fmed.2023.1173291 PMC10233123 37275356

[B4] Direction Générale du Budget (n.d.). Direction Générale Du Budget. Available online at: https://dgb.gov.bf/(Accessed August 2, 2026).

[B5] Kaplan W. A. Laing L. (2003). Paying for pharmaceutical registration in developing countries. Health Policy Plan. 18 (3), 237–248. 10.1093/heapol/czg030 12917265

[B6] Morgan S. G. Yau B. Lumpkin M. M. (2017). The cost of entry: an analysis of pharmaceutical registration fees in low-middle-and high-income countries. PLoS ONE 12 (8), e0182742. 10.1371/journal.pone.0182742 28809931 PMC5557367

[B7] Mullard A. (2011). Mediator scandal rocks French medical community. Lancet 377 (9769), 890–892. 10.1016/S0140-6736(11)60334-6 21409784

[B8] Ndomondo-Sigonda M. Miot J. Naidoo S. Ng’Andu B. Ngum N. Masot N. E. (2020). National medicines regulatory authorities financial sustainability in the East African community. PLOS ONE 15 (7), e0236332. 10.1371/journal.pone.0236332 32702048 PMC7377437

[B9] Owusu-Asante M. Darko D. M. Asamoah-Okyere K. D. Asante-Boateng S. Kermad A. Walker S. (2022). Evaluation of the food and drugs authority, Ghana regulatory review process: challenges and opportunities. Ther. Innovation and Regul. Sci. 57 (2), 372–385. 10.1007/s43441-022-00478-x 36352343 PMC9646276

[B10] Owusu-Asante M. Darko D. M. Semete-Makokotieia B. Adeyeye C. M. Fimbo A. M. Rukwata R. (2025). Regulatory performance of African national medicines regulatory authorities achieving WHO maturity level 3: identifying best practices. Ther. Innov. and Regul. Sci. 60 (1), 260–273. 10.1007/s43441-025-00879-8 41144224 PMC12753557

[B11] Paschke A. Dimancesco D. Vian T. Kohler J. C. Forte G. (2018). Increasing transparency and accountability in national pharmaceutical systems. Bull. World Health Org. 96 (11), 782–791. 10.2471/BLT.17.206516 30455533 PMC6239016

[B12] Rwanda Food and Drugs Authority (n.d.). Rwanda FDA. Available online at: https://rwandafda.gov.rw/(Accessed March 20, 2026).

[B13] Service d’information du Gouvernement (n.d.). Compte Rendu Du Conseil Des Ministres - Service d’information Du Gouvernement. Available online at: https://www.sig.gov.bf/actions-du-gouvernement/compte-rendu-du-conseil-des-ministres (Accessed March 20, 2026).

[B14] Shabani J. B. B. Kayitare E. Nyirimigabo E. Habyalimana V. Murindahabi M. M. Ntirenganya L. (2022). The capacity of young national medicine regulatory authorities to ensure the quality of medicines: case of Rwanda. J. Pharm. Policy Pract. 15 (1), 90. 10.1186/s40545-022-00492-2 36434730 PMC9700871

[B15] Sithole T. Mahlangu G. Salek S. Walker S. (2021). Evaluation of the regulatory review process in Zimbabwe: challenges and opportunities. Ther. Innov. Reg. Sci. 55 (3), 474–489. 10.1007/s43441-020-00242-z 33387356 PMC8021537

[B16] World Health Organisation (2024). WHO Global Benchmarking Tool (GBT) for Evaluation of National Regulatory system of Medical Products: Manual for Benchmarking and Formulation of Institutional Development Plans. Geneva: WHO. Available online at: https://www.who.int/publications/i/item/9789240087637 (Accessed August 2, 2026).

